# Altered tropical seascapes influence patterns of fish assemblage and ecological functions in the Western Indian Ocean

**DOI:** 10.1038/s41598-020-68904-4

**Published:** 2020-07-27

**Authors:** D. H. Chacin, C. D. Stallings, M. Eggertsen, C. Åkerlund, C. Halling, C. Berkström

**Affiliations:** 1 College of Marine Science, University of South Florida, 140 7th Avenue South, St. Petersburg, FL 33701 USA; 2 Gulf Shellfish Institute, 1905 Intermodal Circle, Suite 330, Palmetto, FL 34221 USA; 3 Department of Ecology, Environment, and Plant Sciences, Stockholm University, 106 91 Stockholm, Sweden; 4 Department of Aquatic Resources, Institute of Coastal Research, Swedish University of Agricultural Sciences, Skolgatan 6, 742 42 Öregrund, Sweden

**Keywords:** Ecology, Ecology, Environmental sciences, Ocean sciences

## Abstract

The arrangement and composition of habitats within landscapes and fine-scale habitat characteristics influence community structure and ecological processes. These aspects can be altered by anthropogenic activities, thus influencing associated assemblages. Farming of macroalgae is a common practice in tropical settings and alters the natural composition of seascapes by introducing monoculture patches. The farmed macroalgae may also differ in palatability compared to naturally-occurring macroalgae, influencing herbivory. This study assessed how these farms may differ from natural macroalgal beds in terms of habitat heterogeneity, fish assemblages, and herbivory. We surveyed fish assemblages and deployed macroalgal assays within macroalgal beds, farms and at varying distances from these habitats near Mafia Island, Tanzania. Fish composition and herbivory differed between the habitats likely due to different macrophyte species richness, underlying hard substrate in natural macroalgal beds, and high abundance of browsers nearby the farms. Additionally, fish assemblage patterns and herbivory were not consistent across the seascapes and varied with distance from the focal habitats possibly due to the presence of other habitats. The results suggest alterations of seascapes by farming practices may have consequences on fish assemblages and the ecological functions performed, thus positioning of farms should be carefully considered in management and conservation plans.

## Introduction

The spatial arrangement and composition of habitats within landscapes along with fine-scale habitat characteristics influence patterns of community structure and ecological processes^1–5^. For example, the abundance and diversity of native pollinators (and the process of pollination) in agricultural settings is strongly influenced by spatial characteristics of the landscape, such as the relative proportion of natural habitats neighboring farmed patches^6^. Similarly, in forest settings, organismal fire refuge depends on the composition, fine-scale structure, and spatial arrangement of the vegetation within the landscape^7^. Anthropogenic activities such as land use can influence fine-scale habitat characteristics and the composition of habitats in the landscape, thus affecting associated communities across the globe^8–10^. Landscape alterations can influence habitat heterogeneity, and ultimately food resource quality and availability, resulting in modification of trophic structure, biodiversity, and ecosystem function^11–13^. For instance, agricultural intensification of landscapes has been shown to lower heterogeneity resulting in reduced refuge, feeding areas, and dispersal corridors for birds consequently influencing their diversity, density, and breeding success^14–16^. Land-use patterns can therefore affect the persistence of regional metapopulations and the structure of biological communities^17,18^. This may also be the case in marine systems where alterations of seascape composition and heterogeneity within habitats will likely affect ecosystem functioning.

Farming and harvesting of macroalgae is a common practice in many tropical marine settings ^19,20^. Macroalgal farming has been increasingly advocated as a sustainable alternative form of aquaculture due to lack of fertilizers or medicines applied (e.g., compared to shrimp farms) for many local communities in developing countries and is expanding rapidly worldwide^21–25^. Despite the practice being promoted as a means to improve coral-reef health through poverty reduction and reduced fisheries exploitation^22^, the ecological effects and benefits of macroalgal farming remain unclear, partly because studies have shown contrasting results^26^.

In tropical seascapes, the introduction of macroalgal farms can alter the natural structure and composition of habitats in several ways. Typically, farms consist of numerous ropes arranged in a dense and parallel fashion suspended above the seafloor over large, shallow subtidal areas^26^. Macroalgae thalli are tied to the ropes, introducing habitat patches of monocultures over substrate naturally covered by sand, seagrasses (*Cymodocea* or *Thalassia* spp.), native macroalgae, or small coral patches^27,28^. The macroalgae taxa used in the farming practices may not be native to the farming location, resulting in the introduction of a potentially invasive species, which may spread from the farms into neighboring components of the seascape^29,30^. The farmed macroalgae may also grow faster or differ in palatability to local marine herbivores compared to native macroalgae, resulting in changes in rates of herbivory^31^. The extensive and readily available aggregations of farmed fleshy macroalgae may also attract herbivores within the seascape. All these factors have the potential to influence fish assemblages and the ecological functions the fishes perform, which may depend on the fine-scale heterogeneity within habitats as well as on the arrangement and composition of tropical habitat patches. Therefore, the alteration of tropical seascapes through the introduction of macroalgal farms has the potential to affect the entire ecosystem structure and function.

In contrast to macroalgal farms, beds of native canopy-forming macroalgae occur naturally interspersed within or adjacent to other habitats (e.g., seagrass beds, coral reefs) comprising a mosaic of interlinked patches. Despite the fact that canopy-forming macroalgae in temperate systems has long been recognized as important habitats for fishes^32–35^, tropical natural macroalgal beds have only recently been recognized as important fish habitat offering structural complexity (both through the macroalgae itself and the underlying hard substrate upon which macroalgae grow) and a variety of food resources^36–40^. Natural macroalgal beds in tropical systems can cover extensive areas^41,42^ and enhance productivity by providing habitat and food resources to numerous organisms including fishes and invertebrates^37,38,43–47^. While our knowledge on the ecological role of natural macroalgal beds in tropical seascapes is advancing, information on the process of herbivory (e.g., rates of macroalgae consumption, identification of consumers) within these habitats or on patterns of herbivory and fish assemblages in the surrounding seascape is scarce (but see^47^). Even less is known about how the alteration of seascapes, through the introduction of macroalgal farms, may affect ecological processes that influence ecosystem function and how it compares to seascapes with natural macroalgal beds.

Indeed, macroalgae farming has been demonstrated to influence organismal communities of bacteria^48^, meiofauna^49^, benthic macrofauna^50^, fish^51^, and corals^52^. Macroalgal farms have also influenced fishery catch composition and ecosystem structure and function within seagrass beds^53,54^. However, there are no previous studies in the tropics that compare macroalgal farms to natural macroalgal beds and the surrounding seascape in terms of fish community structure and ecological processes. The goal of this study was to fill this knowledge gap and understand how macroalgal farms differ from naturally-occurring macroalgal beds in terms of fish community assembly and ecological processes such as herbivory. We hypothesized that habitat heterogeneity would be higher in natural macroalgal beds compared to macroalgal farms (due to methods of algae cultivation and underlying substrate) and subsequently fish abundance and richness would be higher in macroalgal beds. We hypothesize that macroalgal farms may attract browsers due to the high concentration of readily available farmed macroalgae in contrast to natural macroalgal beds where macroalgae may be more spread out. Thus, browser abundance as well as the associated macroalgae loss (through herbivory) would be higher in the interior and edge of macroalgal farms compared to natural macroalgal beds and the surrounding seascape. Therefore, we expected macroalgae loss to be context dependent and affected by habitat arrangement and fish assemblages in the seascape.

## Materials and methods

### Study location

**Figure. 1:**
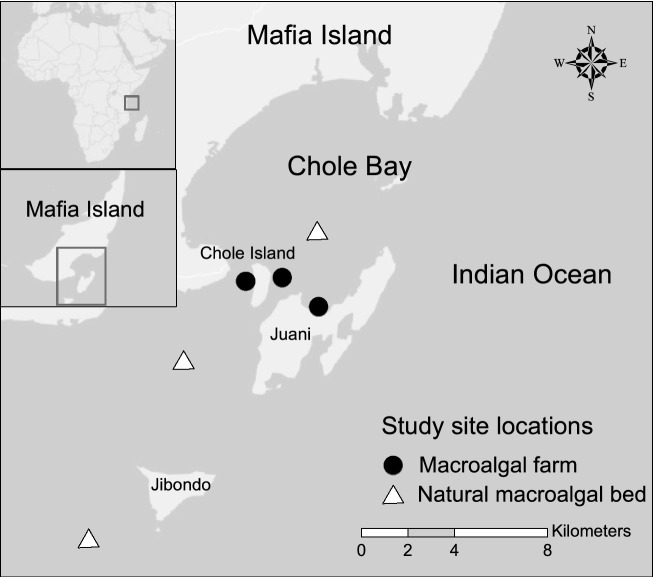
Study sites in Mafia Island, Tanzania. Black circles indicate locations of macroalgal farms and white triangles indicate locations of natural macroalgal beds. Map was generated using ArcMap 10.4.1 (https://desktop.arcgis.com/en/quick-start-guides/10.4/arcgis-desktop-quick-start-guide.htm).

This study was conducted during September–November 2016 in the southern region of Mafia Island off the east coast of Tanzania (Fig. 1). Mafia Island is located 60 km south of Dar es Salaam and 21 km east of the Rufiji delta^55^. The backreef areas around Chole Bay, where the study was conducted, have a maximum depth of 15 m, except in the deep channels that connect the Bay with the open ocean^56^. Mafia Island is influenced by the East African Coastal Current (EACC) and has semidiurnal tides with 3.3 m in average amplitude^55^.

In the tropical Western Indian Ocean, macroalgal farming began on the Zanzibar archipelago, Tanzania in the late 1980s, with the introduction of Philippine strains of *Eucheuma denticulatum* and *Kappaphycus alvarezii* and is currently practiced along the coast, with Zanzibar being the largest producer^57^. These two red macroalgae are farmed in shallow coastal areas for their polysaccharide carrageenan content, which is widely used by the pharmaceutical, food, cosmetics, and textile industries for gelling constituents, medicines, and toothpaste among other products^58^. In addition to tourism, macroalgal farming has become one of the most important industries bringing foreign revenue into the local economy through exports and raising living standards of rural communities^22,23,59^. For this study, we selected *E. denticulatum* as our farmed study species over *K. alvarezii* since its farming is more prevalent in the study area^30,47^.

**Figure 2:**
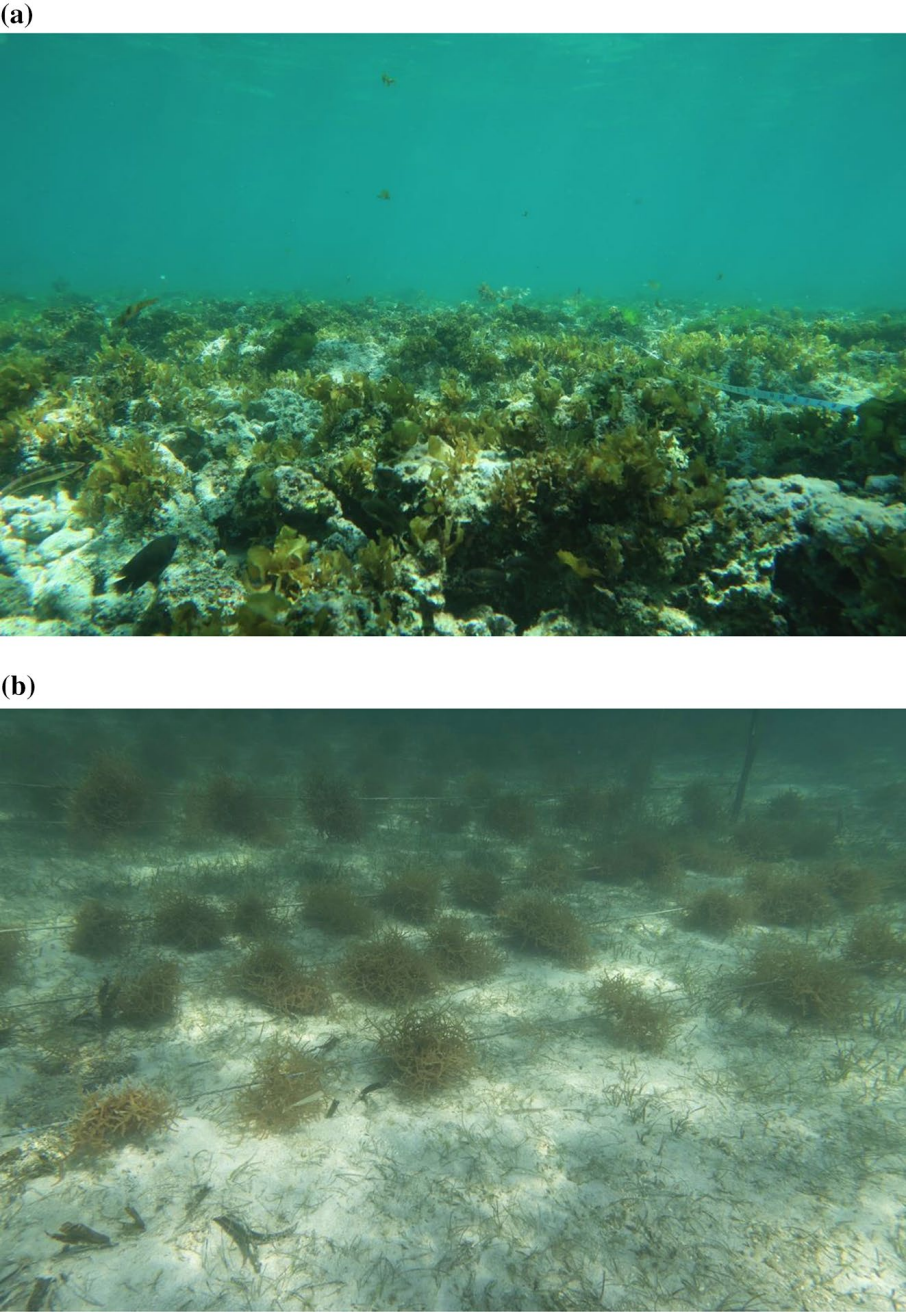
Image of a natural macroalgal bed (**a**) and a macroalgal farm (**b**).

The focal habitats compared in this study included natural macroalgal beds (Fig. 2a), and macroalgal farms (Fig. 2b). The natural macroalgal beds were dominated by *Sargassum aquifolium* and *Turbinaria conoides* with the occasional presence of other species including *Padina* spp., *Portieria harveyi, Ulva* spp*.,* and *E. denticulatum*. The natural macroalgal beds were also heterogeneous with irregular patches of sand, coral, sponges, and rubble mixed in with the macroalgae. Macroalgal farms of *E. denticulatum* were placed in shallow, accessible, and well-flushed coastal areas. The farms require no addition of fertilizers or pesticides, only sufficient water motion and natural light^60,61^). The macroalgae is farmed by using the “peg-and-line” or “off-bottom” method, in which macroalgal fronds are tied using ribbons, known as “tie-ties,” to ropes that are extended and tied to wooden pegs on the marine sediment^61^. The size and spacing of macroalgal fronds was variable, with the size of fronds ranging from ~ 10–30 cm in diameter and tending to be evenly spaced with ~ 40–60 cm of rope in between. Other macrophytes such as seagrasses (*Cymodocea* and *Thalassia* spp.) were occasionally found on the substrate beneath the macroalgae fronds, otherwise sand was the most common substrate. Both the natural macroalgal bed and macroalgal farm sites selected were similar in area (~ 600 m^2^) and seawater temperature (26–28 °C).

The study sites were located within and in the vicinity of Chole Bay, which is an area comprising shallow mosaics of macroalgal beds, seagrass meadows, mangrove shorelines, and coral reefs^62,63^. Three natural macroalgal beds and three macroalgal farms with their surrounding seascape (up to 100 m distance from focal habitat patches) were selected for this study (Fig. 1). We surveyed fish assemblages and conducted field experiments within the focal macroalgal habitats and at varying distances from the habitats in the seascapes to characterize fish assemblages and gain insight into herbivory of the different macroalgae.

### Fish assemblage surveys

To determine whether fish assemblages differed among natural macroalgal beds, macroalgal farms, and the neighboring seascapes, fish surveys (25 m × 2 m strip transects) were conducted in focal macroalgal habitats and at different distances (edge/boundary of focal patches, 10 m, 50 m, and 100 m) from the habitats. This method was selected following the approach used by Tano et al.^38^ and the occasional low visibility within the bay. Fishes were identified to the lowest taxonomic level possible (usually species) and their total lengths were estimated to the closest centimeter. Five minutes after the transect strip was laid down, a snorkeler swam along the transect line ~ 0.1 m s − 1 documenting all mobile fish species and then returned to document small, cryptic species. Sea urchins encountered in our surveys were also included since they are known for their importance as herbivores in benthic marine habitats^64–66^. Surveys were conducted between 0900 and 1630 h during similar tidal height to reduce variability in fish density associated with crepuscular periods. At the end of every survey, benthic substrate composition was categorized (e.g., sand, macroalgae, seagrass, sponges) and quantified within 0.25 m^2^ quadrats every 5 m on the transect line.

We compared fish assemblage structure from field surveys among focal habitats within the seascape. Permutation-based, non-parametric analysis of variance (np-ANOVA) was used to compare fish densities and species richness between the two focal macroalgal habitats and among the different locations in the seascape. Fish assemblages were compared between natural macroalgal beds and macroalgal farms by conducting a permutation-based, non-parametric multivariate analysis of variance^67,68^ (np-MANOVA) on square-root transformed data. This test is a multivariate equivalent of Fisher’s F-statistic and it was employed to test the null hypothesis of no difference among groups^67^. To identify which species were significantly indicative of each group (i.e., macroalgal bed vs. macroalgal farm) the indicator value method^69^ (IndVal) was used. The test was followed by a canonical analysis of principal coordinates^70,71^ (CAP) to create a bi-plot and a correlation vector plot to visualize the main species driving the observed differences. A second CAP bi-plot was produced to visualize herbivore assemblages in natural macroalgal beds and macroalgal farms. Homogeneity of dispersion was verified with the function np-disp, which is equivalent to Levene’s test^72,73^, and when needed data were square-root transformed. All statistical analyses were conducted using MATLAB and the Fathom toolbox^74^.

### Macroalgal assays field experiments

To test potential differences in herbivory among natural macroalgal beds, macroalgal farms, and the surrounding seascapes, a fully-orthogonal field experiment was conducted. Macroalgal species were collected from the field by hand, placed in buckets filled with seawater, and brought back to the laboratory. At the laboratory the macroalgae samples were rinsed multiple times and shaken to remove all large epiphytes. The macroalgae were divided into smaller single-thallus pieces and were spun in a salad spinner for ten seconds to remove any excess water. Single-thallus pieces of *Sargassum aquifolium* and farmed *Eucheuma denticulatum* were tethered within each focal habitat (i.e., macroalgal beds of *Sargassum* spp*.,* macroalgal farms of *E. denticulatum*) for 24 h and at different distances (edge of focal patches, 10 m, 50 m, and 100 m) from each focal habitat to quantify percent of macroalgae loss throughout the surrounding seascape. We hypothesized that the percent loss of the native macroalgae *S. aquifolium* would be higher compared to the farmed *E. denticulatum* due to its higher nutritional content^75^, however this pattern would be context-dependent upon location of deployment.

**Figure 3:**
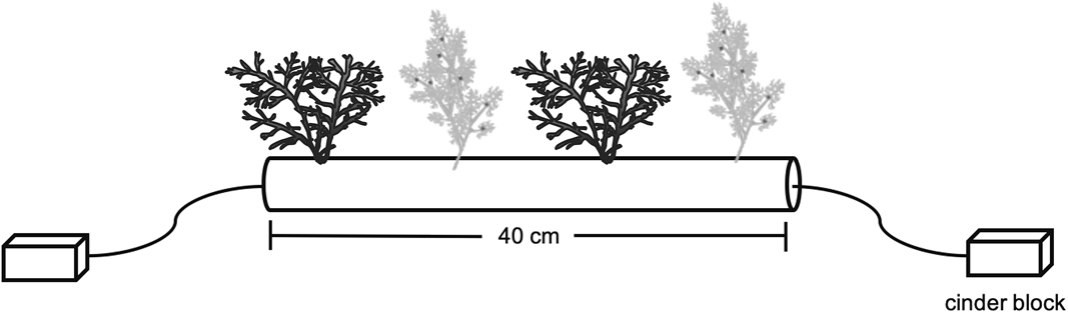
Tethering unit with macroalgae *Eucheuma denticulatum* and *Sargassum aquifolium*. The tethering units were deployed within focal macroalgal patches (macroalgal farms and natural macroalgal beds), at the edges and at 10 m, 50 m, and 100 m from the habitats.

The macroalgal assays were created from a 40 cm long, 3 cm wide PVC pipe where two thalli of each macroalgal species were fixed in place with a transparent plastic line and separated by approximately 8 cm (Fig. 3). For a subset of the macroalgal assays, a video camera (GoPro Hero Session, Black) was placed facing directly toward the assay, and allowed to record for 20 min. At five and ten minutes into the recordings, any fishes that came into the field of view of the camera were noted and identified to the lowest taxonomic level possible. We omitted the first and last five minutes in order to avoid any potential effects related to snorkelers traveling to the camera location thus affecting fish behavior during the set-up and retrieval of the cameras. The remaining time from the videos were further subsampled due to time constraints. The macroalgae were weighted before and after the deployment and percent of macroalgae loss per unit time calculated. The rate of macroalgae loss was then compared between focal macroalgal habitats using permutation-based, non-parametric analysis of variances (np-ANOVAs). To control for the effects of handling or algal growth, a caged control with two thalli pieces of each species was deployed during each sampling event. Macroalgal biomass losses that occurred in the control cages (due to natural reasons) were used in the calculations to adjust experimental (non-control) macroalgae biomass accordingly.

## Results

Habitat heterogeneity was markedly different between natural macroalgal beds and farms while no differences in macrophyte characteristics (i.e. cover, height) were detected between the focal habitats. More specifically, the mean percent cover (± standard error of the mean) of hard substrate (e.g., rubble, rocks, reef) was over four times higher in natural macroalgal beds (39.63 ± 4.89) compared to macroalgal farms (9.42 ± 5.84; *F*_(1, 30)_ = 13.34, *p* = 0.002). The mean macrophyte cover (including seagrass and farmed/non-farmed macroalgae; *F*_(1, 30)_ = 3.18, *p* = 0.08) and height (*F*_(1, 30)_ = 3.18, *p* = 0. 38) were similar between the natural macroalgal beds and macroalgal farms. Although not significant at the alpha 0.05 level, there was a marginal support for higher species richness of macrophytes in natural macroalgal beds (6.4 ± 0.5 species) than in macroalgal farms (4.8 ± 0.4 species; *F*_(1, 30)_ = 4.17, *p* = 0.057).

**Figure 4:**
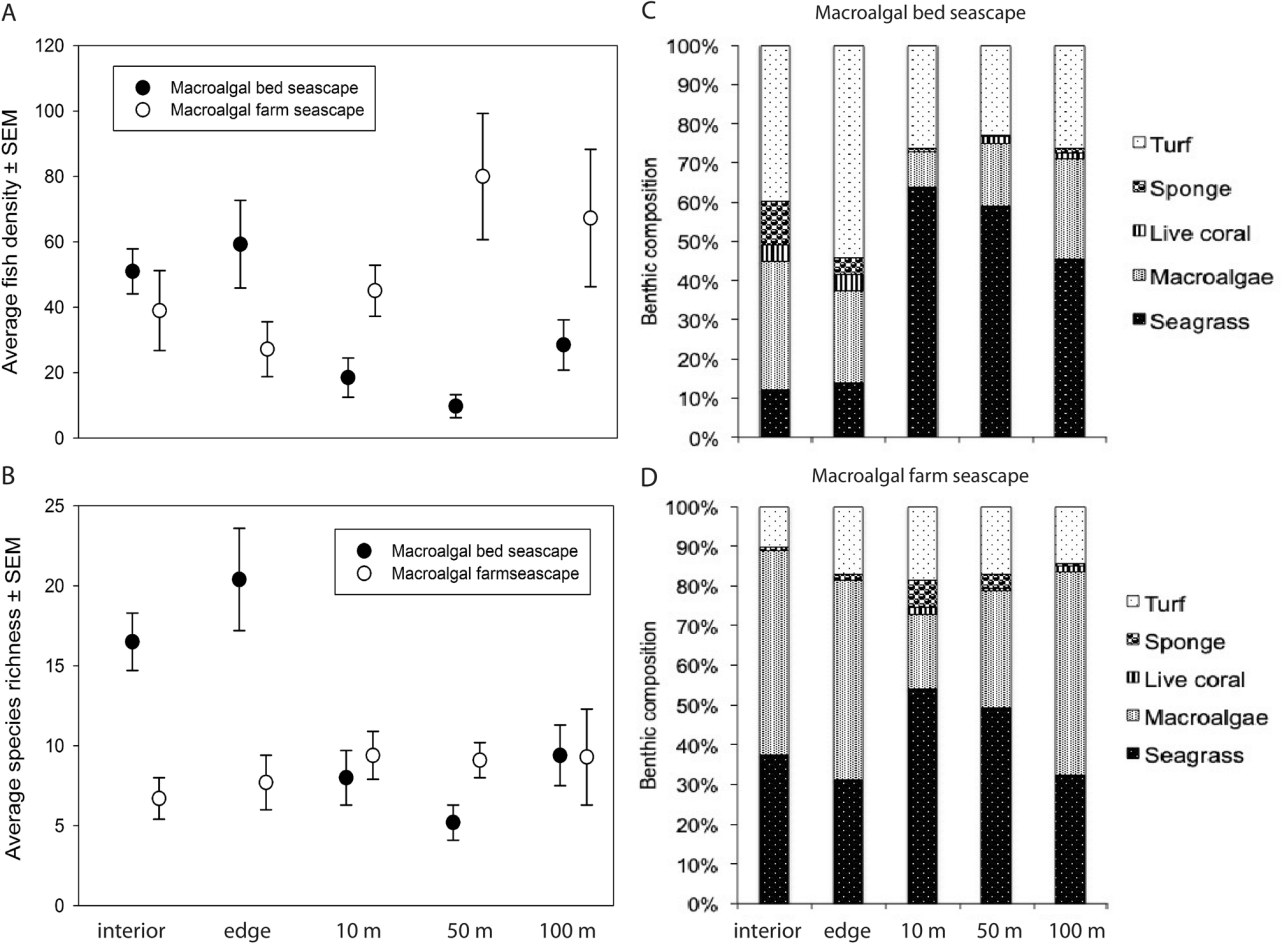
Average (± standard error) fish density (**a**) and species richness (**b**) in natural macroalgal beds, macroalgal farms, and neighboring locations in the seascape. Benthic composition in natural macroalgal beds (**c**) and macroalgal farms (**d**) and neighboring locations within each habitat’s seascape. The term *interior* refers to the interior of the focal habitat, *edge* refers to the boundary of the focal habitat, *10 m* refers to 10 m from the edge of the focal habitat, *50 *m refers to 50 m from the edge of the focal habitat, and *100 m* refers to 100 m from the edge of the focal habitat.

In total, 4,464 fish were quantified from 97 visual surveys (see Supplementary Table S1 online). Fish abundance (mean number of fish per transect ± standard error of the mean) was similar between the interior of focal habitats, with 51.0 ± 6.9 fish observed in natural macroalgal beds and 39.0 ± 12.2 fish in macroalgal farms (*F*_(1, 30)_ = 2.15, *p* = 0.09). Fish abundance did not differ between the interior and edge of natural macroalgal beds (*t* = 0.50, *p* = 0.722) but decreased significantly with distance away from the natural macroalgal beds (*F*_(4, 43)_ = 4.42, *p* =  < 0.01; Fig. 4a). In contrast, fish abundance tended to increase with distance away from farms in the seascape (Fig. 4a). However, this pattern was not statistically significant (*F*_(4, 42)_ = 1.78, *p* = 0.06).

Fish species richness (± standard error of the mean) was higher in natural macroalgal beds (16.5 ± 1.8 fish species) than in macroalgal farms (6.7 ± 1.3 fish species; *F*_(1, 30)_ = 10.7, *p* =  < 0.01). Species richness decreased drastically within 10 m from the edge of natural macroalgal beds and stayed rather constant at greater distances away from the edge of natural macroalgal beds (*F*_(4,43)_ = 5.52, *p* =  < 0.01; Fig. 4b). Species richness in macroalgal farms, on the other hand, was constant throughout the seascape (*F*_(4, 42)_ = 1.78, *p* = 0.07; Fig. 4b).

On average, a greater percentage of turf, sponges, and macroalgae were observed in the interior and edge of natural macroalgal beds while the percentage of seagrass (dominated by *Thalassodendron ciliatum*) increased with distance away from the focal habitat (Fig. 4c). In seascapes with macroalgal farms, macroalgae and seagrass beds were the most common benthic substrate while turf and sponges constituted comparatively lower percentages (Fig. 4d). The seagrasses *Cymodocea* spp. and *Thalassia hemprichii* were the dominant species in the interior and edge of macroalgal farms, while the seagrass *Enhalus acoroides* dominated the seagrass composition in the surrounding seascape.

**Figure 5:**
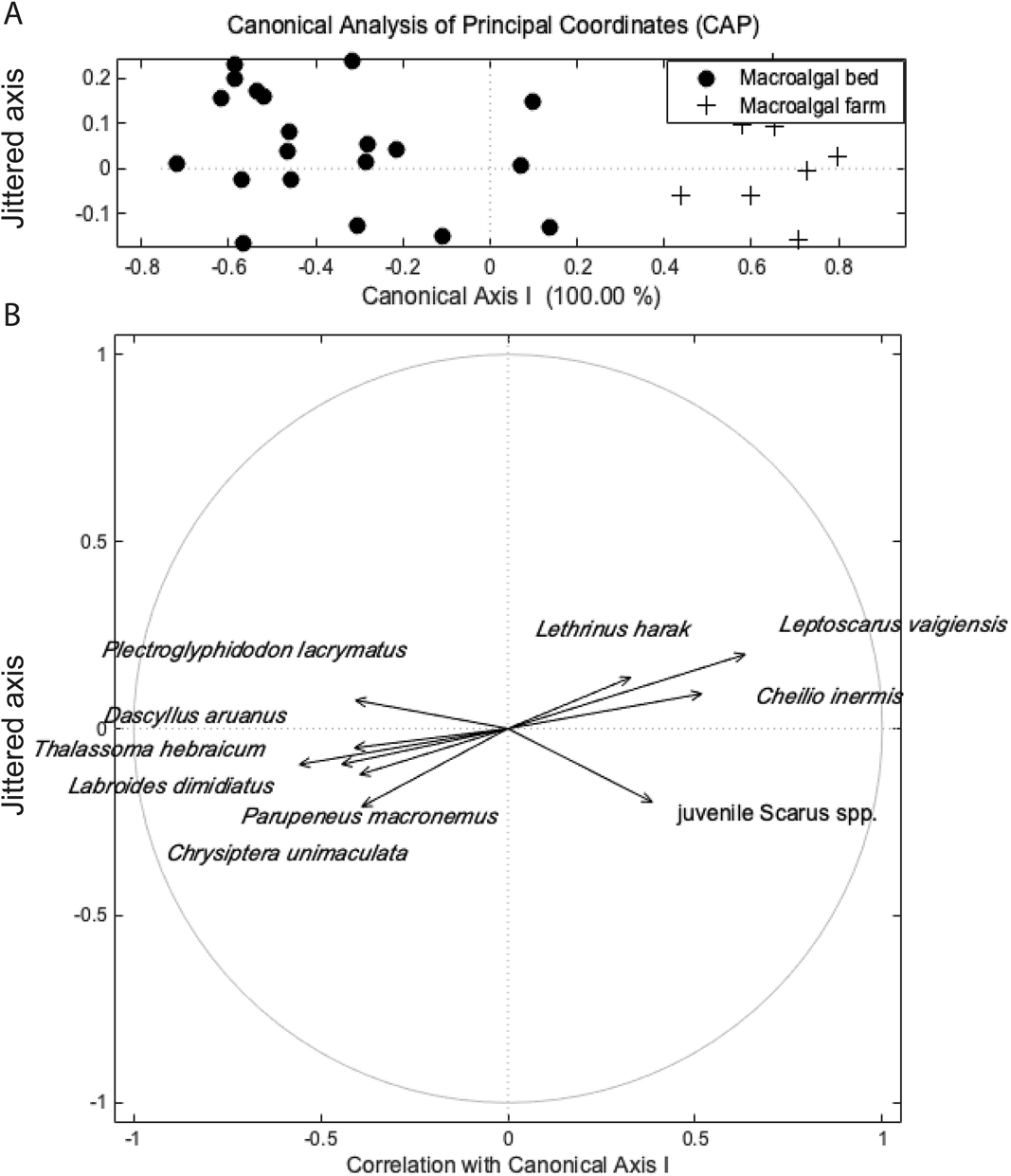
Canonical Analysis of Principal Coordinates of Mafia Island, Tanzania fish assemblage structure (**a**). Circles represent data points in natural macroalgal beds and crosses represent data points in macroalgal farms. The Y-axis data are jittered to ease visual assessment of the assemblage patterns. Fish species vectors pointing to the right correspond to those species mainly found in macroalgal farms and those vectors pointing to the left correspond to those observed in the macroalgal beds (**b**).

Fish assemblage composition differed significantly between natural macroalgal beds and farms (*F*_(1, 30)_ = 5.33 , *p* = 0.001). The ten species with highest indicator power values were identified and visualized with a CAP plot (Table 1). The CAP demonstrated through the correlation vectors with the longest components on canonical axis I, that wrasses such as *Thalassoma hebraicum, Labroides dimidiatus*, the goatfish *Parupeneus macronemus*, and damselfishes *Plectroglyphidodon lacrymatus, Dascyllus aruanus,* and *Chrysiptera unimaculata* were the taxa most indicative of the macroalgal beds driving the assemblage patterns. Parrotfishes such as *Leptoscarus vaigensis* and juvenile Scarus spp., the wrasse *Cheilio inermis,* and the emperor *Lethrinus harak* were the taxa most indicative of macroalgal farms (Fig. 5). A second CAP plot with focus on the herbivore assemblage, showed similar results in that the browsers *L. vaigiensis, Siganus sutor* and juvenile Scarus species were common in the macroalgal farms, while grazers, scrapers, and territorial grazers such as *Chrysiptera unimaculata*, *Scarus ghobban* and *Stegastes nigricans* respectively, drove assemblage patterns in natural macroalgal beds (see Supplementary Fig. S1 online).

**Table 1:** Results of IndVal analysis comparing natural macroalgal beds versus macroalgal farms. The 10 species with highest indicator power values (ranges from 0–100%) were selected and used for visualization in the CAP plot.

Group	Family	Taxa	*I*	*p-*value
Macroalgal bed	Labridae (wrasses parrotfishes)	*Thalassoma hebraicum*	76.19	0.001
	Labridae (wrasses parrotfishes)	*Labroides dimidiatus*	57.14	0.007
	Pomacentridae (damselfishes)	*Plectroglyphidodon lacrymatus*	52.38	0.013
	Pomacentridae (damselfishes)	*Chrysiptera unimaculata*	46.44	0.057
	Pomacentridae (damselfishes)	*Dascyllus aruanus*	42.86	0.022
	Mullidae (goatfishes)	*Parupeneus macronemus*	38.85	0.034
Macroalgal farm	Labridae (wrasses and parrotfishes)	*Leptoscarus vaigiensis*	70.10	0.002
	Labridae (wrasses and parrotfishes)	Juvenile Scarus spp.	49.67	0.066
	Labridae (wrasses parrotfishes)	*Cheilio inermis*	43.74	0.015
	Lethrinidae (emperors)	*Lethrinus harak*	27.27	0.025

**Figure 6:**
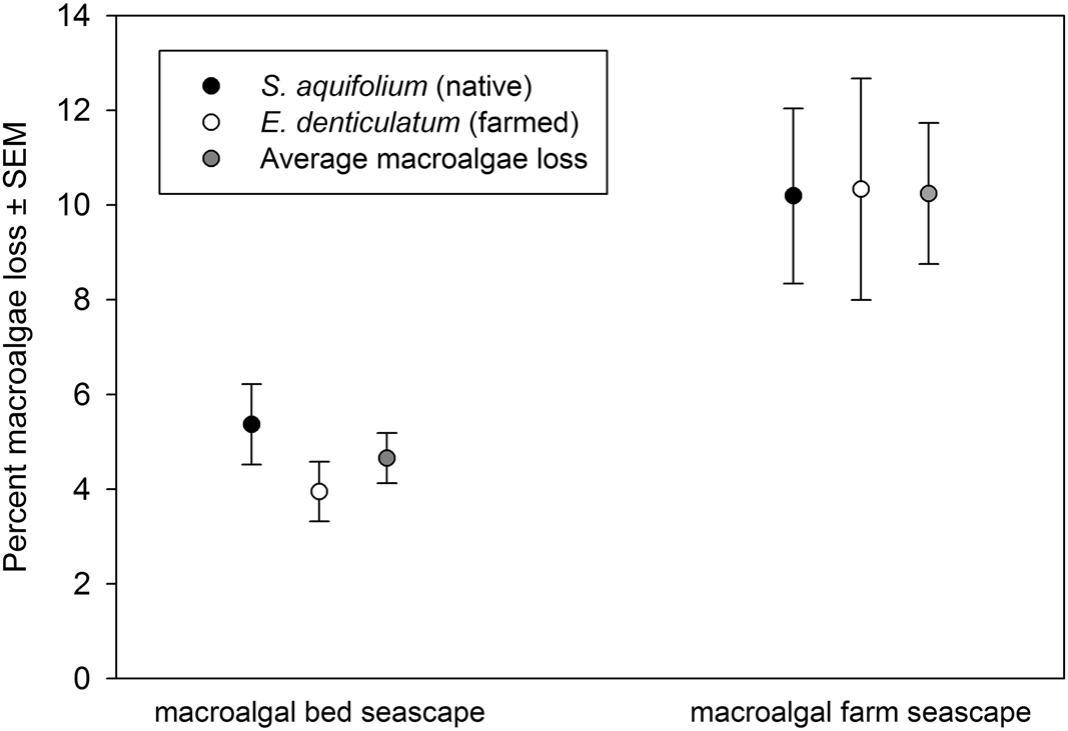
Percent loss of macroalgae in macroalgal bed seascapes and macroalgal farm seascapes ± standard error of the mean.

**Figure 7:**
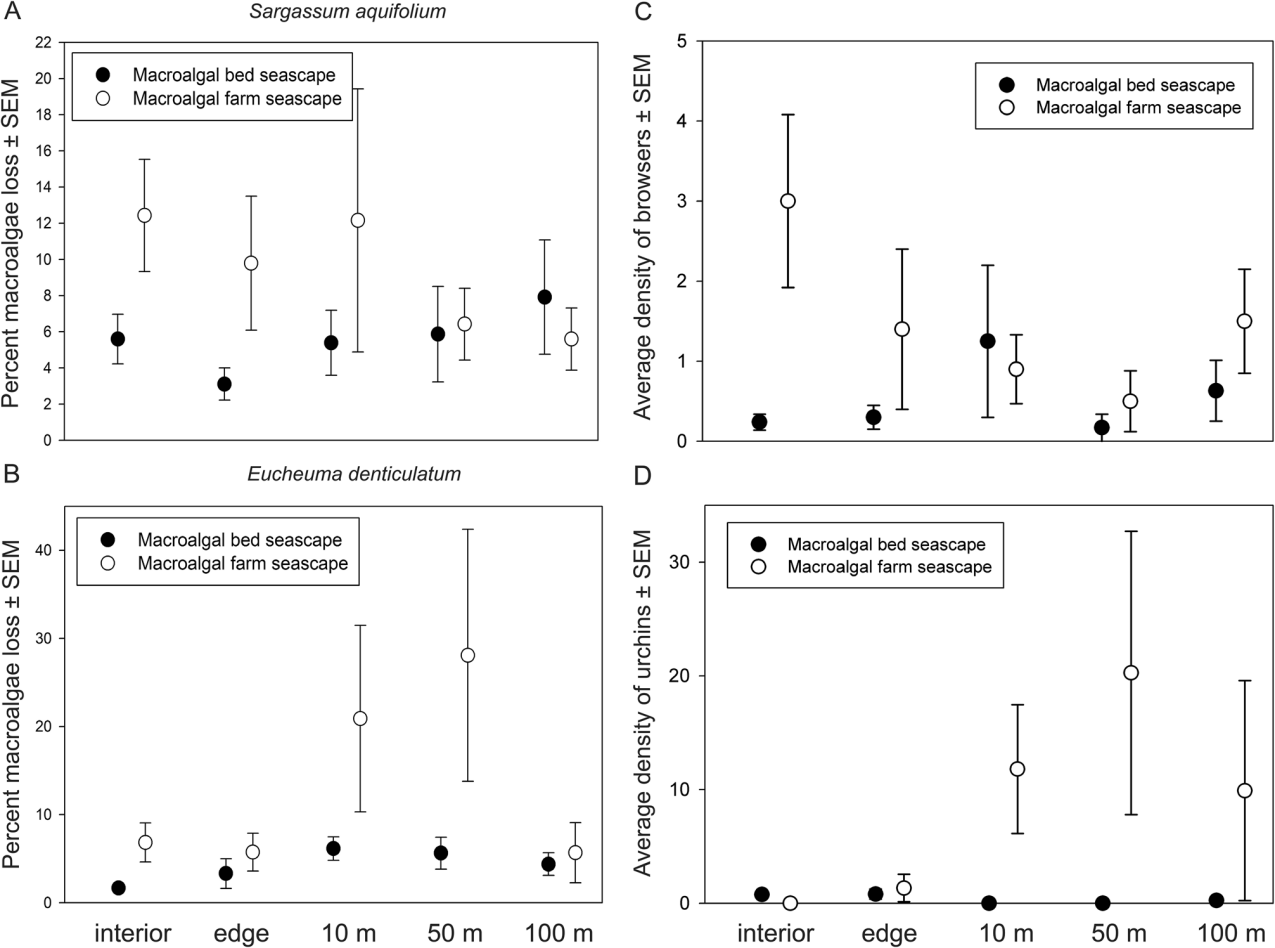
Percent loss (mean ± standard error) of *Sargassum aquifolium* (**a**) and *Eucheuma denticulatum* (**b**) in natural macroalgal beds, macroalgal farms, and neighboring locations in the seascape. Average density (± standard error) of browsers (**c**) and sea urchins (**d**) in natural macroalgal beds, macroalgal farms, and neighboring locations in the seascape. The term *interior* refers to the interior of the focal habitat, *edge* refers to the boundary of the focal habitat, *10 m* refers to 10 m from the edge of the focal habitat, *50 m* refers to 50 m from the edge of the focal habitat, and *100 m* refers to 100 m from the edge of the focal habitat.

In general, macroalgal loss during the macroalgae assay field experiments was higher and more variable in macroalgal farm seascapes compared to macroalgal bed seascapes (*F*_(1, 386)_ = 14.60 , *p* = 0.001; Fig. 6). Furthermore, percent loss of *S. aquifolium* did not differ from *E. denticulatum* in the seascapes for either natural macroalgal beds (*F*_(1, 212)_ = 1.79 , *p* = 0.21; Fig. 6) or farms (*F*_(1, 172)_ = 0.002 , *p* = 0.953). There was no difference in mean loss of *S. aquifolium* among the different distances away from natural macroalgal beds (*F*_(4,102)_ = 0.84, *p* = 0.53; Fig. 7a) or among distances away from the macroalgal farms (*F*_(4,82)_ = 0.42, *p* = 0.82; Fig. 7a). The percent loss of *E. denticulatum* did not differ among the different distances in the natural macroalgal beds (*F*_(4,102)_ = 1.75, *p* = 0.131; Fig. 7b). In addition to higher percentage of *E. denticulatum* loss in the macroalgal farms compared to natural macroalgal beds, the percentage of macroalgae loss was both higher and more variable at 10 and 50 m from the farms compared to the other locations within the seascape (*F*_(4,82)_ = 3.01, *p* = 0.02; Fig. 7b).

In terms of trophic composition, the surveys demonstrated that territorial grazers, other grazers, and scrapers were common in the interior and edge of natural macroalgal beds, and the percentage of territorial grazers decreased in the surrounding seascape (Fig. S2a). In the interior and edge of macroalgal farms, a greater percentage of browsers was present compared to the surrounding seascape wherein the percentages of browsers decreased with increasing distance from farms (Fig. S2b). Instead, the percentage of scrapers and excavators increased in the surrounding seascape compared to the interior and edge of the macroalgal farms (Fig. S2b). Furthermore, the abundance of browsers was also higher in the interior and edge of macroalgal farms, and both lower and more consistent in natural macroalgal beds and neighboring seascape (Fig. 7c). Sea urchins were nearly absent from natural macroalgal bed seascapes and not abundant in the interior and edge of the farms (Fig. 7d). Sea urchin abundance was higher and more variable in the neighboring seascape of the macroalgal farms (Fig. 7d). Invertivores in contrast, were more common in natural macroalgal beds and the surrounding seascape compared to the macroalgal farms and the surrounding seascape (see Supplementary Fig. S2 online).

## Discussion

As hypothesized, patterns of fish assemblage, habitat heterogeneity, and percent of macroalgae loss differed between natural macroalgal beds and macroalgal farms in tropical Western Indian Ocean seascapes. Additionally, fish assemblages and rates of herbivory were not consistent across the seascapes and varied with distance from the focal habitat patches likely due to the presence of other benthic habitats in the seascape. However, contrary to predictions, certain macrophyte characteristics (such as cover and height) did not differ and fish abundance was not higher in natural macroalgal beds compared to macroalgal farms. The results of the present study indicate that macroalgal farms, such as those of monospecific *Eucheuma denticulatum*, do not host the same fish assemblage as natural macroalgal beds and that the underlying substratum in natural macroalgal beds could have been an important factor influencing fish assemblages. Similar to Bergman et al.^55^ and Anyango et al.^76^, macroalgal farms also differed in fish trophic composition from surrounding uncultivated habitats (e.g., seagrass beds, macroalgal patches) in the seascape and had a high abundance of browsers. This suggests alterations of tropical seascapes by farming practices may have consequences on fish community composition and the ecological functions performed.

Fish abundance was similar among natural macroalgal beds and macroalgal farms and may be explained by similar habitat characteristics and food resource availability among habitats. Habitat characteristics such as macrophyte cover and height, which provide structural complexity in ecosystems, were similar between natural macroalgal beds and macroalgal farms. The dense structure of macrophytes can impede effective foraging by predators, consequently reducing predation rates^77^ and macroalgal density, height, and percent cover have been identified as important drivers of fish abundance and distribution in temperate reefs^34,78^. Similarly, canopy height, density of holdfasts, and coverage of macroalgal habitat patches are important predictors of fish assemblages (e.g., settlement, abundance) in the Western Indian Ocean as well as other tropical locations such as in the Caribbean and Indo-Pacific^36,39,47,79–81^. Additionally, high seagrass blade density (which is usually correlated with macrophyte cover) and the presence of macroalgae can reduce mortality rates of fishes^82,83^ and many invertebrates^84–87^, suggesting that the structural complexity provided by the two macroalgal habitats in Mafia Island likely offered similar refuge to fishes.

Fish species richness was, however, higher in natural macroalgal beds compared to macroalgal farms and both habitats hosted different fish assemblages, which may have been related to the individual or combined effects of higher macrophyte species richness and greater percentage cover of underlying hard substrate found in natural macroalgal beds. The combination of different macrophyte species and hard substrate in natural macroalgal beds likely provided fine-scale habitat heterogeneity, thus increasing structural complexity and potentially greater food and habitat resources for fishes^43^. Indeed, the interior of natural macroalgal beds had higher cover of sponges, corals, and filamentous algal turf (compared to the interior of the macroalgal farms), and these may have offered crevices and holes for a variety of species to use as refuge^47^. Similarly, van Lier et al.^46^ observed that hard complexity coupled with soft canopy structure of tropical natural macroalgal beds in Western Australia provided a large number of microhabitat types, and thus supported a high number of species likely due to niche partitioning^88,89^. Similar patterns have been observed in cichlid fish communities in lakes in central Africa and in Iberian bat populations where habitat heterogeneity allows partitioning of resources and supports diverse assemblages^90,91^. This is likely the reason why the territorial pomacentrid *P. lacrymatus,* which farms and feeds on the epilithic algal matrix^92^ growing on hard substrates^93^, and the invertivores *T. hebraicum and P. macronemus*, which feed on invertebrates found on macroalgae and hard substrates, were found to highly influence assemblage patterns in natural macroalgal beds. Moreover, monocultures on land in agricultural fields can have lower within-habitat heterogeneity and lower species richness of associated organisms compared to natural, uncultivated vegetative habitats^94,95^. Similar patterns could have occurred in the macroalgal farms where monocultures offer less structural complexity and variety of resources thus lowering fish species richness. Overall, fine-scale heterogeneity due to both macroalgae and underlying hard substrate likely influenced assemblage patterns, but we were not able to disentangle one factor from the other. Future experimental studies that orthogonally manipulate and control the presence and amount of macroalgae and hard substrate would improve our understanding of the relative importance of each of these factors for fish assemblages.

In contrast, the macroalgal farms had lower percentage of hard substrate and filamentous algal turf, and were mainly composed of macroalgae and seagrass. This is likely the reason why macroalgal farms comprised a different fish assemblage with higher percentage of herbivorous fishes and a lower percentage of carnivores, omnivores, and corallivores compared to natural macroalgal beds. Similar results have been observed in terrestrial agronomic practices where for instance, ground beetle assemblages in uncultivated fields differed from those in harvested fields^96^. In the present study the parrotfish *L. vaigiensis*, and juvenile Scarus spp. were the most abundant herbivores and some of the most indicative taxa in macroalgal farms. The high presence of the parrotfish *L. vaigiensis* in farms may be explained by its feeding ecology. It feeds on both algae and seagrass blades and may do so both within the farms and in the nearby surrounding seagrass patches^97^. Other studies have observed high abundances of siganids (rabbitfishes—common browsers in Indo-Pacific tropical seascapes) within macroalgal farms in Zanzibar, Tanzania^51,54,76^. Surprisingly, siganids were recorded in low numbers in the visual surveys. However, the rabbitfish *Siganus sutor* was frequently observed swimming in 43% of the deployed videos in the macroalgal farms, suggesting this species evades human presence and the reason why we did not observe them frequently in our visual surveys. Although siganids are targeted by artisanal fishers in the study region, possibly explaining their avoidance behaviors towards human presence, similar observations have been made on the Great Barrier Reef where they are not fished^98,99^, suggesting general avoidance behavior to divers. In Southeast Asia (Indonesia, Malaysia, and the Philippines), siganid catches have increased disproportionally with macroalgal farming^26^. While it is unclear whether a similar increase in siganid abundance is occurring in the tropical Western Indian Ocean, where farming is performed at a substantially smaller-scale, it is not surprising to observe the siganids swimming in and out the macroalgal farms. All the siganids observed in the videos were juveniles, which could indicate recruitment to these macroalgal habitats similar to patterns observed in other tropical macroalgal locations^81^. Overall, these patterns suggest that while both macroalgal habitats (natural beds and farms) may provide similar structural complexity for fishes (to some extent), the identity of the macroalgae and the available amount of hard structural complexity can influence the composition of the assemblage, determining the ecological functions performed by the fishes.

Fish abundance and species richness in the surrounding seascape differed between natural macroalgal beds and macroalgal farms. In seascapes where natural macroalgal beds were located, fish abundance and species richness were similar between the interior and edge of the focal habitat, and generally decreased with distance from the habitat. These patterns were likely influenced by the change of benthic composition from structurally diverse habitats including turf and sponges to a benthic cover of the monospecific *Thalassodendron ciliatum* seagrass, resulting in reduced habitat heterogeneity as distance increased away from natural macroalgal beds. As previously discussed, high fine-scale habitat heterogeneity offers a range of resources, both in terms of refuge and feeding grounds, hence attracting a more diverse fish assemblage^100,101^. In addition, lower epiphytic cover in seagrass beds compared to those of other benthic habitats in tropical settings may also provide less food resources and thus lower abundance and richness of associated fish. In contrast, both epiphytic growth on seagrass and fish abundance is high in subtropical and temperate seagrass beds^102,103^. The abundance and species richness of fishes was similar across the seascape where macroalgal farms were located, even as distance increased from the focal habitat. Other types of benthic habitats present in the vicinity of the farms likely influenced these patterns. Patches of seagrasses mainly dominated by the long-bladed *Enhalus acoroides* may have provided refuge for many fishes similar to the mix of seagrass and algae in the macroalgal farms, thus explaining the consistent pattern of fish species richness across the seascape surrounding the farms^104^. High fish density and diversity has previously been associated with patches of *E. acoroides* since several taxa are able to rely on *E. acoroides* as a food source^104,105^.

Macroalgae loss due to herbivores was generally higher in seascapes where macroalgal farms were located compared to seascapes where natural macroalgal beds were present. These patterns were likely related to the greater number of browsers in seascapes with farms. Indeed, abundance of browsers was higher inside the macroalgal farms suggesting the readily available farmed *E. denticulatum* served as a viable food source. Additionally, *E. denticulatum* was intensively grazed while under cultivation in the present study and has been found in the stomachs of browsers (e.g., *Siganus* spp*.*) in the tropical Western Indian Ocean^51,76^. It was also the most frequently found macroalgae in the stomachs of the rabbitfish *Siganus sutor*, caught in the same macroalgal farms as the present study^75^. Comparably, in terrestrial farmlands granivorous bird species (i.e., those with a substantial seed component in the diet) can feed off cereal grain and the seeds of many plants cultivated in the farmlands and thus benefit from these harvestable habitats^106^.

Biomass loss of *E. denticulatum* and *S. aquifolium* was low in the natural macroalgal beds and did not differ significantly in the surrounding seascape. These patterns were likely related to the low percentage of browsers and low abundance of grazing urchins in the surrounding seascape. Instead, invertivores and omnivores constituted a high percentage of the fishes observed in the surrounding seascapes of natural macroalgal beds. Tanzanian natural macroalgal beds comprise a high abundance and diversity of epifaunal invertebrates^31^, thus these habitats can serve as feeding grounds, not only for herbivorous fishes, but also for a number of invertivores and omnivores.

In contrast to the surrounding seascape of natural macroalgal beds, the percent loss of *E. denticulatum* was not consistent across the seascape with macroalgal farms. It was lower in the interior and edge of the farms compared to the rest of the seascape despite the high abundance of browsers observed. This may be explained by the high availability of *E. denticulatum* fronds in the farms, resulting in lower browsing intensity on the tethered *E. denticulatum*. Similar patterns have been described in other studies^107,108^. Alternatively, some herbivorous fish may avoid feeding in densely aggregated macroalgal areas^109,110^ such as within the farms. The biomass loss of *E. denticulatum* was higher and more variable with distance from the farms despite the decrease in abundance of browsers and might have been related to less available farmed macroalgae as a food source in the surrounding seascape. It may also have been influenced by the sporadically high abundance of grazing urchins found in the vicinity of the farms. Interestingly, patchy aggregations of sea urchins, including *Diadema savigni*, *D. setosum*, *Echinometra mathaei*, and *Echinotrix diadema* were encountered in the vicinity of the farms but not within them. Occasionally, during low tide, the macroalgal farms were exposed to the air and thus unavailable as feeding grounds for marine herbivores. Air exposure can be detrimental to marine invertebrates due to desiccation stress^111^. This is possibly the reason why the sea urchins were found nearby the farms and not in their interior and why the percent loss of macroalgae in general tended to be more variable in the vicinity of the farms.

In this study we observed that macroalgal farms differed from naturally-occurring macroalgal beds, both in terms of habitat heterogenity and fish species richness. This suggests that macroalgal farming may modify habitat heterogeneity of coastal habitats with potential secondary effects on community assemblage and ecological processes. If the intensity of the macroalgal farming were to increase (such as in Southeast Asia), it could lead to homogenization and degradation of coastal habitats across the seascape with losses in the diversity of macrophytes and fishes with consequences on ecosystem function. In agricultural terrestrial systems increased land use intensity has caused loss of habitat heterogeneity resulting in declines of species richness of birds and arthropods^16,112–116^. As such, the placement of farms and farming intensity might have significant implications for associated fauna and ecological processes. Alternatively, macroalgae farming if practiced properly could have positive effects, such as carbon sequestration to mitigate carbon emissions^117^. Therefore, further studies are recommended to understand generalities. This study allowed us to gain a greater understanding of how “land-use” practices in seascapes may influence patterns of community assembly and ecological processes and was an attempt to attain a more holistic view of tropical seascapes in the Western Indian Ocean subjected to human practices.

## Supplementary information

Supplementary file1 (DOCX 23 kb)

Supplementary file2 (DOCX 192 kb)

Supplementary file3 (DOCX 442 kb)
