# Supplementary material for: Altered tropical seascapes influence patterns of fish assemblage and ecological functions in the Western Indian Ocean
Source: Sci Rep. 2020 Jul 27;10:12479. doi: 10.1038/s41598-020-68904-4 (PMC7385177; doi:10.1038/s41598-020-68904-4)
Supplement: Supplementary file 1 — Supplementary file1 (DOCX 23 kb) [file 41598_2020_68904_MOESM1_ESM.docx]

**Altered tropical seascapes influence patterns of fish assemblage and ecological functions in the Western Indian Ocean**

Chacin, D.H.; Stallings, C.D.; Eggertsen, M.; Åkerlund, C.; Halling, C.; Berkström, C.

Table S1. Mean abundance ± standard error of all fish and urchin species observed during visual surveys in both habitats. Letters in parenthesis indicate functional group: B: browser, G: grazer, E: excavator, TG: territorial grazer (farmer), D: detritivore, O: omnivore, I: invertivore, C: corallivore, P: piscivore, S: scraper.

| Species | Macroalgal bed | Macroalgal farm |
| --- | --- | --- |
| *Abudefduf sexfasciatus* (OG) | 0.00 ± 0.00 | 2.18 ± 1.56 |
| *Abudefduf sparoides* (O) | 0.14 ± 0.10 | 0.45 ± 0.37 |
| *Abudefduf vaigiensis* (OG) | 0.05 ± 0.05 | 0.00 ± 0.00 |
| *Acanthuridae spp* (G) | 0.05 ± 0.05 | 0.09 ± 0.09 |
| *Acanthurus nigrofuscus* (G) | 0.14 ± 0.10 | 0.00 ± 0.00 |
| *Acanthurus triostegus* (G) | 0.24 ± 0.19 | 0.00 ± 0.00 |
| *Amphiprion akallopisos* (G) | 0.24 ± 0.15 | 0.00 ± 0.00 |
| *Amphiprion allardi* (G) | 0.86 ± 0.30 | 0.00 ± 0.00 |
| *Anampses meleagrides* (C) | 0.05 ± 0.05 | 0.00 ± 0.00 |
| *Apogon cookie* (I) | 0.29 ± 0.14 | 0.00 ± 0.00 |
| *Apogon cyanosoma* (I) | 0.05 ± 0.05 | 0.00 ± 0.00 |
| *Apogonichthyoides taeniatus* (I) | 0.00 ± 0.00 | 0.55 ± 0.39 |
| *Arothron caeruleopunctatus* (I) | 0.05 ± 0.05 | 0.00 ± 0.00 |
| *Balistoides viredescens* (I) | 0.10 ± 0.10 | 0.00 ± 0.00 |
| *Callionymidae* (I) | 0.05 ± 0.05 | 0.00 ± 0.00 |
| *Calotomus carolinus* (B) | 0.14 ± 0.08 | 0.00 ± 0.00 |
| *Canthigaster bennetti* (O) | 0.52 ± 0.22 | 0.27 ± 0.20 |
| *Canthigaster solandri* (O) | 0.33 ± 0.17 | 0.00 ± 0.00 |
| *Canthigaster valentini* (O) | 0.62 ± 0.26 | 0.18 ± 0.12 |
| *Centropyge multispinis* (G) | 0.57 ± 0.27 | 0.00 ± 0.00 |
| *Cetoscarus bicolor* (E) | 0.14 ± 0.10 | 0.00 ± 0.00 |
| *Chaetodon auriga* (O) | 0.38 ± 0.16 | 0.00 ± 0.00 |
| *Chaetodon kleinii* (O) | 0.05 ± 0.05 | 0.00 ± 0.00 |
| *Chaetodon melannotus* (C) | 0.05 ± 0.05 | 0.00 ± 0.00 |
| *Chaetodon trifascialis* (C) | 0.05 ± 0.20 | 0.00 ± 0.00 |
| *Chaetodon trifasciatus* (C) | 0.29 ± 0.10 | 0.00 ± 0.00 |
| *Chaetodon xanthocephalus* (O) | 0.14 ± 0.05 | 0.00 ± 0.00 |
| *Cheilinus chlorourus* (I) | 0.05 ± 0.10 | 0.00 ± 0.00 |
| *Cheilinus oxycephalus* (PI) | 0.10 ± 0.10 | 0.00 ± 0.00 |
| *Cheilinus trilobatus* (PI) | 0.00 ± 0.00 | 0.09 ± 0.09 |
| *Cheilinus undulatus* (PI) | 0.00 ± 0.00 | 0.09 ± 0.09 |
| *Cheilio inermis* (PI) | 0.19 ± 0.11 | 0.82 ± 0.26 |
| *Cheilodipterus macrodon* (PI) | 0.05 ± 0.05 | 0.00 ± 0.00 |
| *Cheilodipterus quinquelineatus* (PI) | 0.48 ± 0.24 | 2.00 ± 1.64 |
| *Chlorurus sordidus* (E) | 0.14 ± 0.14 | 0.00 ± 0.00 |
| *Chromis fieldi* (I) | 0.19 ± 0.13 | 0.00 ± 0.00 |
| *Chromis nigrura* (I) | 0.05 ± 0.05 | 0.00 ± 0.00 |
| *Chromis viridis* (I) | 1.05 ± 1.05 | 0.00 ± 0.00 |
| *Chromis weberii* (I) | 0.81 ± 0.43 | 0.00 ± 0.00 |
| *Chrysiptera annulata* (O) | 0.43 ± 0.26 | 0.00 ± 0.00 |
| *Chrysiptera biocellata* (G) | 0.81 ± 0.31 | 0.27 ± 0.19 |
| *Chrysiptera unimaculata* (G) | 1.90 ± 0.61 | 0.27 ± 0.19 |
| *Cirrhitichthys oxycephalus* (I) | 0.19 ± 0.09 | 0.00 ± 0.00 |
| *Coris caudimacula* (I) | 0.52 ± 0.25 | 0.09 ± 0.09 |
| *Coris cuvieri* (I) | 0.24 ± 0.12 | 0.00 ± 0.00 |
| *Coris Formosa* (I) | 0.14 ± 0.08 | 0.00 ± 0.00 |
| *Cryptocentrus lutheri* (I) | 0.33 ± 0.14 | 0.73 ± 0.38 |
| *Ctenochaetus binotatus* (GD) | 0.05 ± 0.05 | 0.00 ± 0.00 |
| *Dascyllus aruanus* (O) | 3.71 ± 1.34 | 0.00 ± 0.00 |
| *Dascyllus reticulatus* (O) | 1.10 ± 0.83 | 0.00 ± 0.00 |
| *Dascyllus trimaculatus* (O) | 2.10 ± 1.06 | 0.64 ± 0.64 |
| *Diadema setosum* (G) | 0.14 ± 0.14 | 0.00 ± 0.00 |
| *Diodon liturosus* (I) | 0.14 ± 0.08 | 0.00 ± 0.00 |
| *Echinometra mathaei* (G) | 0.38 ± 0.21 | 0.00 ± 0.00 |
| *Echinotrix diadema* (G) | 0.24 ± 0.12 | 0.00 ± 0.00 |
| *Epinephelus tauvina* (PI) | 0.10 ± 0.07 | 0.00 ± 0.00 |
| *Epinephelus fasciatus* (PI) | 0.14 ± 0.10 | 0.00 ± 0.00 |
| *Epinephelus merra* (PI) | 0.10 ± 0.10 | 0.00 ± 0.00 |
| *Fistularia commersonii* (PI) | 0.05 ± 0.05 | 0.00 ± 0.00 |
| *Gnatholepis cauerensis* (O) | 0.19 ± 0.11 | 2.00 ± 1.43 |
| *Gobiidae spp.* (I) | 0.05 ± 0.05 | 0.00 ± 0.00 |
| *Gomphosus caeruleus* (I) | 0.52 ± 0.21 | 0.00 ± 0.00 |
| *Gymnothorax flavimarginatus* (PI) | 0.10 ± 0.07 | 0.00 ± 0.00 |
| *Gymnothorax javanicus* (PI) | 0.05 ± 0.05 | 0.00 ± 0.00 |
| *Gymnothorax richardsonii* (PI) | 0.00 ± 0.00 | 0.09 ± 0.09 |
| *Halichoeres hortulanus* (I) | 0.48 ± 0.13 | 0.09 ± 0.09 |
| *Halichoeres scapularis* (I) | 0.90 ± 0.32 | 1.00 ± 0.75 |
| *Halichoeres zeylonicus* (I) | 0.00 ± 0.00 | 0.09 ± 0.09 |
| *Istigobius decoratus* (I) | 0.05 ± 0.05 | 0.00 ± 0.00 |
| *Labroides dimidiatus* (I) | 1.48 ± 0.00 | 0.00 ± 0.00 |
| *Lactoria cornuta* (I) | 0.00 ± 0.00 | 0.09 ± 0.09 |
| *Leptoscarus vaigiensis* (B) | 0.05 ± 0.05 | 2.55 ± 0.85 |
| *Lethrinus harak* (I) | 0.00 ± 0.00 | 2.27 ± 1.81 |
| *Lutjanus fulviflamma* (PI) | 0.00 ± 0.00 | 0.27 ± 0.27 |
| *Macropharyngodon bipartitus* (I) | 0.05 ± 0.05 | 0.00 ± 0.00 |
| *Meiacanthus mossambicus* (O) | 0.29 ± 0.12 | 0.00 ± 0.00 |
| *Monotaxis grandoculis* (I) | 0.10 ± 0.07 | 0.00 ± 0.00 |
| *Muraenidae* (PI) | 0.05 ± 0.05 | 0.00 ± 0.00 |
| *Myrichthys colubrinus* (PI) | 0.00 ± 0.00 | 0.09 ± 0.09 |
| *Neoniphon argenteus* (PI) | 0.10 ± 0.10 | 0.09 ± 0.09 |
| *Neoniphon samara* (I) | 0.00 ± 0.00 | 0.09 ± 0.09 |
| *Novaculichthys taeniourus* (I) | 0.14 ± 0.08 | 0.00 ± 0.00 |
| *Ostorhinchus cookie* (I) | 0.14 ± 0.10 | 0.00 ± 0.00 |
| *Ostorhinchus cyanosoma* (I) | 0.05 ± 0.05 | 0.00 ± 0.00 |
| *Oxycheilinus bimaculatus* (I) | 0.05 ± 0.05 | 0.00 ± 0.00 |
| *Papilloculiceps longiceps* (P) | 0.05 ± 0.05 | 0.00 ± 0.00 |
| *Paraluteres prionurus* (O) | 0.14 ± 0.14 | 0.00 ± 0.00 |
| *Parapercis hexophtalma* (I) | 0.19 ± 0.11 | 0.00 ± 0.00 |
| *Parupeneus macronemus* (I) | 1.00 ± 0.35 | 0.00 ± 0.00 |
| *Pervagor janthinosoma* (O) | 0.05 ± 0.05 | 0.00 ± 0.00 |
| *Plectroglyphidodon lacrymatus* (TG) | 3.33 ± 1.11 | 0.00 ± 0.00 |
| *Plotosus lineatus* (O) | 0.43 ± 0.43 | 2.73 ± 2.73 |
| *Pomacentrus baenschi* (I) | 0.38 ± 0.16 | 0.00 ± 0.00 |
| *Pomacentrus caeruleus* (O) | 0.48 ± 0.21 | 0.00 ± 0.00 |
| *Pomacentrus pavo* (O) | 0.29 ± 0.29 | 0.00 ± 0.00 |
| *Pomacentrus sulfureus* (I) | 0.24 ± 0.24 | 0.00 ± 0.00 |
| *Pseudocheilinus hexataenia* (I) | 0.33 ± 0.21 | 0.00 ± 0.00 |
| *Pteragogus flagellifera* (I) | 0.29 ± 0.16 | 0.09 ± 0.09 |
| *Sargocentron diadema* (I) | 0.76 ± 0.28 | 0.00 ± 0.00 |
| *Saurida gracilis* (PI) | 0.00 ± 0.00 | 0.18 ± 0.12 |
| *Scarinae spp* (S) | 4.76 ± 1.67 | 15.91 ± 5.47 |
| *Scarus frenatus* (S) | 2.14 ± 1.42 | 0.00 ± 0.00 |
| *Scarus ghobban* (S) | 1.62 ± 1.06 | 0.27 ± 0.27 |
| *Scarus rubroviolaceus* (S) | 0.19 ± 0.11 | 0.00 ± 0.00 |
| *Scarus scaber* (S) | 0.00 ± 0.00 | 0.09 ± 0.09 |
| *Scolopsis ghanam* (PI) | 0.52 ± 0.31 | 0.09 ± 0.09 |
| *Siganus luridus* (B) | 0.00 ± 0.00 | 0.18 ± 0.18 |
| *Siganus sutor* (B) | 0.05 ± 0.05 | 0.27 ± 0.19 |
| *Stegastes nigricans* (TG) | 2.14 ± 1.18 | 0.00 ± 0.00 |
| *Stethojulis albovittata* (I) | 2.05 ± 0.47 | 1.45 ± 0.56 |
| *Stethojulis strigiventer* (DI) | 0.00 ± 0.00 | 0.18 ± 0.18 |
| *Synodus binotatus* (PI) | 0.19 ± 0.11 | 0.00 ± 0.00 |
| *Taeniura lymma* (PI) | 0.00 ± 0.00 | 0.09 ± 0.09 |
| *Thalassoma amblycephalum* (I) | 0.14 ± 0.10 | 0.00 ± 0.00 |
| *Thalassoma Hardwicke* (I) | 0.33 ± 0.19 | 0.00 ± 0.00 |
| *Thalassoma hebraicum* (I) | 2.38 ± 0.51 | 0.00 ± 0.00 |
| *Thalassoma lunare* (I) | 0.29 ± 0.14 | 0.00 ± 0.00 |
