# Supplementary material for: Altered tropical seascapes influence patterns of fish assemblage and ecological functions in the Western Indian Ocean
Source: Sci Rep. 2020 Jul 27;10:12479. doi: 10.1038/s41598-020-68904-4 (PMC7385177; doi:10.1038/s41598-020-68904-4)
Supplement: Supplementary file 2 — Supplementary file2 (DOCX 192 kb) [file 41598_2020_68904_MOESM2_ESM.docx]

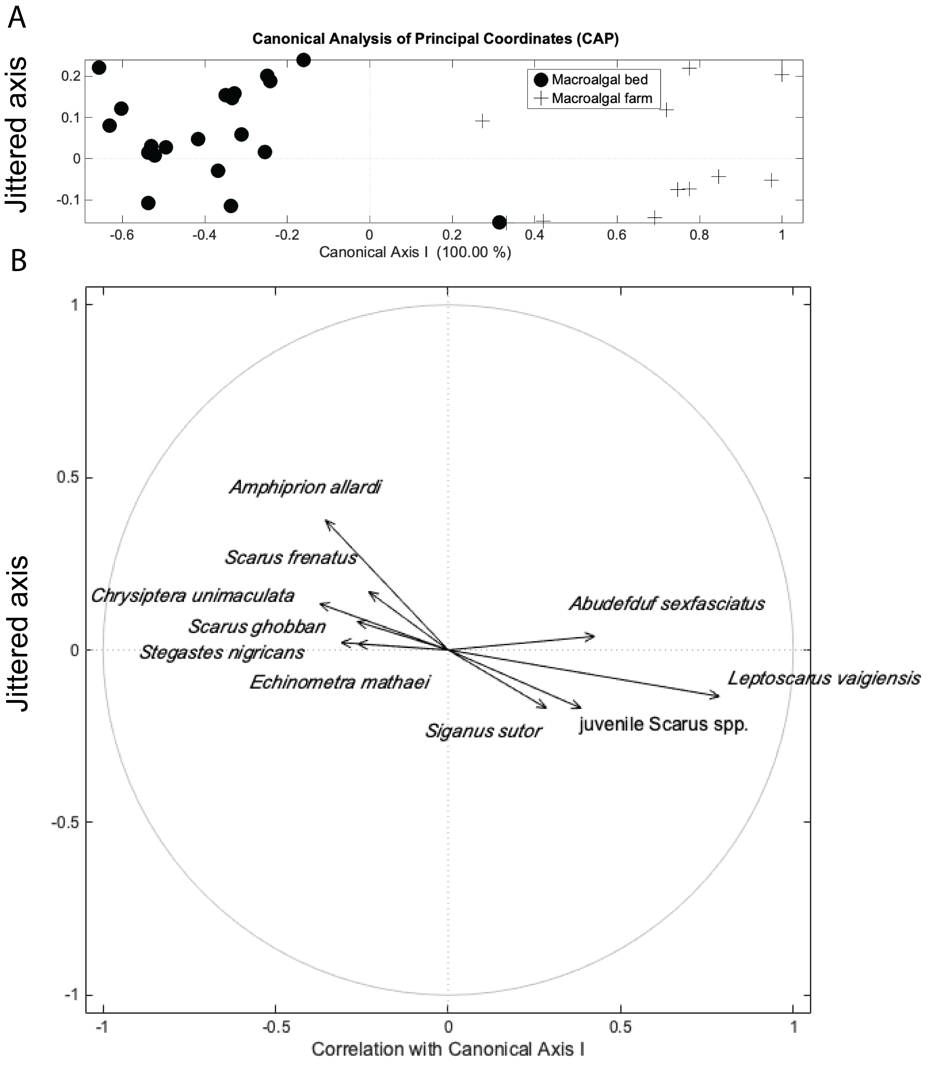


Figure S1. Canonical Analysis of Principal Coordinates of Mafia Island, Tanzania herbivore assemblage (a). Circles represent data points in natural macroalgal beds and crosses represent data points in macroalgal farms. The Y-axis data are jittered to ease visual assessment of the assemblage patterns. Fish species vectors pointing to the right correspond to those species mainly found in macroalgal farms and those vectors pointing to the left correspond to those observed in the natural macroalgal beds (b).
