# Supplementary material for: Altered tropical seascapes influence patterns of fish assemblage and ecological functions in the Western Indian Ocean
Source: Sci Rep. 2020 Jul 27;10:12479. doi: 10.1038/s41598-020-68904-4 (PMC7385177; doi:10.1038/s41598-020-68904-4)
Supplement: Supplementary file 3 — Supplementary file3 (DOCX 442 kb) [file 41598_2020_68904_MOESM3_ESM.docx]

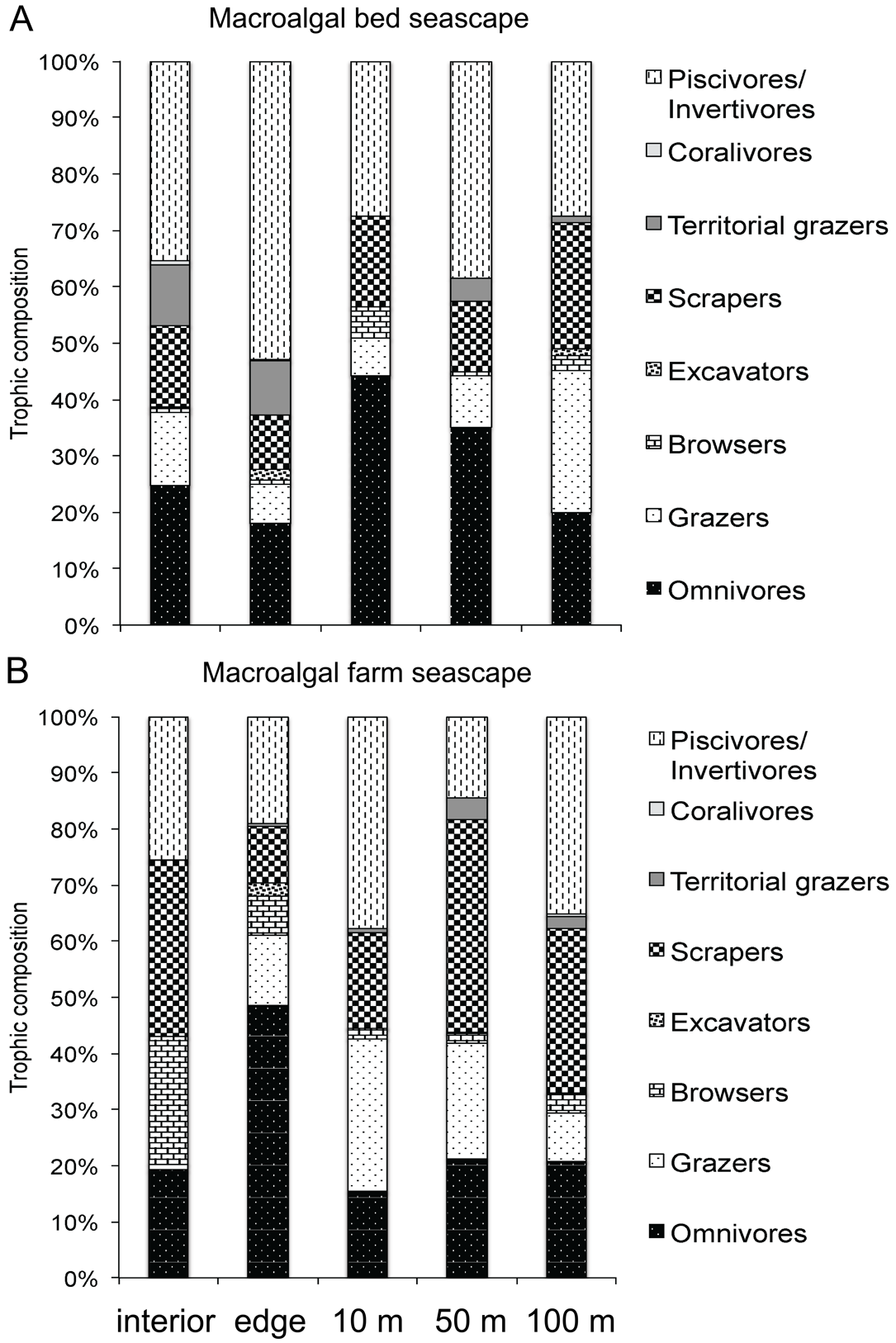


Figure S2. Trophic composition in natural macroalgal beds (a) and macroalgal farms (b) and neighboring locations within the seascape of each habitat. The term *interior* refers to the interior of the focal habitat, *edge* refers to the boundary of the focal habitat, *10 m* refers to 10 meters from the edge of the focal habitat, *50 m* refers to 50 meters from the edge of the focal habitat, and *100 m* refers to 100 meters from the edge of the focal habitat.
